# Higher-order aberrations and their association with axial elongation in highly myopic children and adolescents

**DOI:** 10.1136/bjophthalmol-2021-319769

**Published:** 2022-01-13

**Authors:** Yupeng Xu, Junjie Deng, Bo Zhang, Xian Xu, Tianyu Cheng, Jingjing Wang, Shuyu Xiong, Mengli Luan, Haidong Zou, Xiangui He, Chun Tang, Xun Xu

**Affiliations:** 1 Shanghai Eye Disease Prevention and Treatment Center, Shanghai Eye Hospital, Shanghai, China; 2 Department of Ophthalmology, Department of Ophthalmology, Shanghai General Hospital, Shanghai Jiao Tong University School of Medicine, National Clinical Research Center for Eye Diseases, Shanghai Key Laboratory of Ocular Fundus Diseases, Shanghai Engineering Center for Visual Science and Photomedicine, Shanghai Engineering Center for Precise Diagnosis and Treatment of Eye Diseases, Shanghai, China

**Keywords:** epidemiology, ocular surface, optics and refraction, public health, visual pathway

## Abstract

**Background:**

Vision-dependent mechanisms play a role in myopia progression in childhood. Thus, we investigated the distribution of ocular and corneal higher-order aberrations (HOAs) in highly myopic Chinese children and adolescents and the relationship between HOA components and 1-year axial eye growth.

**Methods:**

Baseline cycloplegic ocular and corneal HOAs, axial length (AL), spherical equivalent (SE), astigmatism and interpupillary distance (IPD) were determined for the right eyes of 458 highly myopic (SE ≤−5.0D) subjects. HOAs were compared among baseline age groups (≤12 years, 13–15 years and 16–18 years). Ninety-nine subjects completed the 1-year follow-up. Linear mixed model analyses were applied to determine the association between HOA components, other known confounding variables (age, gender, SE, astigmatism and IPD) and axial growth. A comparison with data from an early study of moderate myopia were conducted.

**Results:**

Almost all ocular HOAs and few corneal HOAs exhibited significant differences between different age groups (all p<0.05). After 1 year, only ocular HOA components was significantly negative associated with a longer AL, including secondary horizontal comatic aberration (p=0.019), primary spherical aberration (p<0.001) and spherical HOA (p=0.026). Comparing with the moderate myopia data, the association of comatic aberration with AL growth was only found in high myopia.

**Conclusion:**

In highly myopic children and adolescents, lower levels of annual ocular secondary horizontal comatic aberration changes, besides spherical aberrations, were associated with axial elongation. This suggests that ocular HOA plays a potential role in refractive development in high myopia.

## Introduction

Myopia is a global health concern and the second most common cause of blindness worldwide.^1 2^ Its prevalence has been on a rapid upward trajectory, especially in East Asia.^3^ Due to the lack of effective therapy, it has been estimated that approximately half of the world’s population could suffer from myopia by 2050. A total of 1/10th of this population will present with high myopia.^4^ Individuals with high myopia that is associated with axial length (AL) elongation are prone to visual complications such as cataracts, glaucoma, macular disease, peripapillary deformation and posterior staphyloma.^5^ The aetiology of myopia has been linked to the interactions of multiple environmental and genetic risk factors.^1 6^ To date, factors that slow down the rapid progression to high myopia have not been fully elucidated.

Vision-dependent mechanisms may play a vital role in the emmetropization process and refractive error development in childhood.^7^ The degradation of images projected onto the retina by ocular aberrations influences visual quality.^8^ Lower-order aberrations such as defocus and astigmatism can be rectified using traditional optical corrections (spectacles or contact lenses). However, optical imperfections described as higher-order aberrations (HOAs) cannot be rectified using these methods. Thus, HOAs provide retinal cues that contribute to the development of the eye.^9^ Studies have documented the relationships between HOA and components of refractive status, such as refractive error (including astigmatism),^10 11^ AL,^12–14^ interpupillary distance (IPD),^12–14^ age^15–17^ and ethnicity.^18^ Greater levels of spherical and comatic aberrations were associated with a longer AL but slower axial elongation.^13 14^ These non-interventional observational studies highlight the potential role of specific habitual HOAs in regulating eye growth and myopia progression in childhood.

However, the association of HOA with the progression of high myopia in children and adolescents is still unclear, as the myopia progression characteristics in non-high myopia and high myopia are different, as presented in the early study of our team.^19^ In young highly myopic population, the start age of significantly declined lens power was 1 year earlier than that reported in previous studies with non-high myopic subjects.^20^ This implied that ocular growth as well as lens power loss launched earlier in high myopes than non-high myopes.^19 21^

Therefore, the aim of this study was to determine the distribution of ocular and corneal HOA characteristics in highly myopic young Chinese children and to examine the potential impact of HOA changes on axial elongation over a 1-year period while controlling for known confounding variables.

## Materials and methods

### Study design

This was a retrospective cohort study. A total of 458 children and adolescents with high myopia (spherical equivalent (SE) ≤−5D) aged between 6 and 18 years were recruited at approximately the same time. The measure of sample size was mainly described in our previous study.^19^ With-the-rule astigmatism (negative cylinders 180°±20°) with best-corrected monocular visual acuity ≤0.10 (Logarithm of Mininal Angle Resolution (logMAR)) were recruited to represent the majority.^22^ Participants with organic eye diseases, including amblyopia, strabismus, down syndrome, moderate-severe ptosis, congenital cataract, glaucoma and the use of contact lenses or orthokeratology, were excluded, and those who were unable to complete all examinations were not included in the final analysis. All parents and guardians of those enrolled in the study were presented with a written informed consent form for signing.

### Study participants and examination procedures

All participants were subjected to a detailed visual examination to confirm normal ocular conditions. Participants with a visual acuity of less than 0.10 logMAR or with ocular pathologies were excluded during the recruitment stage. For cycloplegic eye examination, participants were administered 1 drop of 0.5% proparacaine, 1% tropicamide and 1% cyclopentolate 5 min apart.^12 23 24^ Cycloplegic subjective refraction was performed under lighting conditions according to the principle of maximum plus for maximum visual acuity. A year later, 99 children within the same cohort were enrolled, and the same measurements were performed. None of these children had any visual acuity loss or gain during the 1-year follow-up.

AL measurements were performed using a IOLMaster (Carl Zeiss Meditec AG., Yena, Germany) according to the manufacturer’s instructions. An average of at least five measurements was used for analysis. This device has good repeatability of AL measurement in cycloplegic subjects with ages and refractive errors similar to those of the participants in our study.^25^ Ocular and corneal HOAs for a 5 mm pupil were measured using a Shack-Hartmann aberrometer (Visionix, Luneau Technologies, Chartres, France) after cycloplegia, as dilation and mild cycloplegia did not clinically affect the wave measurement magnitude or pattern.^11 24 26 27^ The obtained data were fitted with a sixth-order Zernike polynomial using a fixed 5 mm pupil diameter. Five measurement repetitions were performed for each eye, and the three best-focused results were averaged for analysis.^14 28^ During measurements, room illumination was kept to a minimum to reduce the impact of stray light.^14^ Zernike coefficients and the root mean square (RMS; in micrometres) of ocular and corneal total, spherical, comatic and trefoil aberrations (total: from third-order to sixth-order terms; spherical aberrations: $Z_{4}^{0}$ and $Z_{6}^{0}$ combined; comatic aberrations: $Z_{3}^{-1},Z_{3}^{1},Z_{5}^{-1},$ and $Z_{5}^{1}$ combined; trefoil aberrations: $Z_{3}^{-3},Z_{3}^{3},Z_{5}^{-3}$ and $Z_{5}^{3}$ ) were computed to indicate wavefront aberrations.^12 29 30^ Strehl ratio(SR) of ocular and corneal total aberrations was computed from the measured wavefront aberrations for a pupil diameter of 5 mm according to the equation below 31. The aberrations were calculated at a reference wavelength (λ) of 555 nm.

$$SR=e^{-{\left(\frac{2\pi }{\lambda }RMS\right)}^{2}}$$

Where RMS is the root mean square of ocular and corneal total aberrations.

During the 1-year follow-up, calibration of the IOLMaster and Shack-Hartmann aberrometer were checked as described in previous studies.^13 14 24^ To reduce the influence of mirror symmetry between eyes and statistical errors in the analysis, data from only the right eyes were obtained.^32^

### Statistical analysis

Statistical analyses were performed using SPSS V.25 (IBM). Statistical significance was set at p≤0.05. For continuous variables, data are presented as mean ± SD. Distributional normality was tested between different groups. The heterogeneity of variance was detected using Levene’s test, and assuming unequal variances, intergroup differences were determined by t-test with Bonferroni correction.^28 33^ Comparisons of SE, AL, SR and HOAs among the three age groups (primary school ≤12 years, middle school 13–15 years and high school 16–18 years) were performed using two-way analysis of variance. Gender proportions were compared using the χ^2^ test. When normality of distribution was not attained, the Mann-Whitney U test was used in the comparison of 1-year changes between different genders. The paired t-test was used to compare baseline and 1-year follow-ups. An early study of moderate myopia was compared with the baseline data of our follow-up cohort using the t test with Bonferroni correction.

Regarding the influence of the changes in HOA components on axial elongation, age was transformed (using a natural logarithm) to fit the models.^24^ To account for random and sporadic missing data in the follow-ups, a linear mixed model was applied to investigate the effect of HOA components on axial elongation. Adjustments for other predictive variables (age, gender, SE, astigmatism and IPD) were performed to control for their influence on axial elongation. The model used a first-order autoregressive covariance structure and restricted maximum likelihood estimation. The fitness of the model was presented as R^2^.

## Results

### Demographics and HOAs between sex and age in highly myopic subjects at baseline

A total of 458 children and adolescents aged between 6 and 18 years were enrolled in this study. Their mean age was 13.51±2.48 years with a mean SE of −8.50±1.74 D, a mean astigmatism of −1.61±1.04 D, a mean IPD of 61.28±3.56 mm and a mean AL of 26.79±1.00 mm (table 1).

**Table 1:** Demographics of the pooled population and differences among different age groups at baseline

Parameters	Total	Age group (years)			P value†
		≤12	13–15	16–18	
Age, years	13.51±2.48	10.64±1.50	13.94±0.80	16.71±0.76	<0.001*
No	458	146	209	103	
Gender, boys%	45.60	41.78	44.98	52.43	0.245
SE, D	−8.50±1.74	−8.13±1.88	−8.52±1.67	−8.95±1.58	0.012*
AL, mm	26.79±1.00	26.42±0.94	26.94±1.01	27.02±0.94	<0.001*
BCVA, logMAR	0.01±0.04	0.02±0.04	0.01±0.04	0.01±0.05	0.154
IPD, mm	61.28±3.56	59.46±3.33	61.82±3.22	62.75±3.53	<0.001*
Astigmatism, D	−1.61±1.04	−1.61±1.05	−1.57±1.01	−1.69±1.09	0.733

The χ^2^ test was used for the comparison of the proportion of gender.

The t-tests with Bonferroni Correction was used for the comparison between subgroups.

The bold values were those P values <0.05.

*P<0.05 was considered statistically significant.

†The analysis of variance test was used for the comparison among three age groups.

‡LogMar means Logarithm of Mininal Angle Resolution

AL, axial length; BCVA, best-corrected visual acuity; D, dioptres; HOA, higher-order aberration; IPD, inter pupillary distance; SE, spherical equivalent.

The ocular total HOA RMS of the whole study group was 0.25±0.11 µm, in which spherical, comatic and trefoil HOA RMS were 0.06±0.05 µm, 0.18±0.11 µm and 0.11±0.07 µm, respectively. Meanwhile, the corneal total HOA RMS of the whole study group was 0.27±0.10 µm, in which the spherical, comatic and trefoil HOA RMS was 0.10±0.05 µm, 0.15±0.09 µm and 0.14±0.08 µm, respectively. The Strehl ratio of ocular and corneal HOA was 0.03±0.09 and 0.01±0.05 (table 2). The RMS of individual ocular and corneal HOA were analysed in online supplemental table 1.

10.1136/bjophthalmol-2021-319769.supp1 Supplementary data

**Table 2:** Ocular and corneal HOAs of pooled population and difference among different age groups at baseline

Parameters	Total	Age, years			P value†
	(n=458)	≤12 (n=146)	13–15 (n=209)	16–18 (n=103)	
Ocular, μm					
Total HOA RMS	0.25±0.11	0.25±0.10	0.24±0.10	0.27±0.13	0.091
SR	0.03±0.09	0.03±0.07	0.04±0.11	0.02±0.06	0.342
Spherical HOA RMS	0.06±0.05	0.06±0.05	0.06±0.05	0.06±0.05	0.622
$Z_{4}^{0}$ ‡	0.01±0.08	0.00±0.08	0.02±0.07	0.02±0.08	0.011*
$Z_{6}^{0}$ ‡	−0.01±0.01	−0.01±0.01	−0.01±0.01	0.00±0.01	<0.001*
Comatic HOA RMS	0.18±0.11	0.18±0.10	0.18±0.11	0.20±0.13	0.307
$Z_{3}^{-1}$	0.12±0.14	0.12±0.14	0.12±0.14	0.13±0.17	0.620
$Z_{3}^{1}$ ‡	0.00±0.09	0.00±0.09	−0.01±0.09	0.03±0.09	0.005*
$Z_{5}^{-1}$	0.01±0.02	0.01±0.02	0.02±0.02	0.02±0.03	0.038*
$Z_{5}^{1}$	0.00±0.01	0.00±0.01	0.00±0.01	0.00±0.01	0.013*
Trefoil HOA RMS	0.11±0.07	0.11±0.06	0.11±0.06	0.13±0.08	0.042*
$Z_{3}^{-3}$ ‡§	−0.02±0.10	0.00±0.10	−0.02±0.09	−0.06±0.11	<0.001*
$Z_{3}^{3}$ §	−0.02±0.08	−0.03±0.08	−0.02±0.08	0.00±0.08	0.018*
$Z_{5}^{-3}$ §	0.00±0.02	0.01±0.02	0.00±0.02	0.00±0.02	0.001*
$Z_{5}^{3}$	0.00±0.01	0.00±0.01	0.00±0.01	0.00±0.01	0.068
Corneal, μm					
Total HOA RMS	0.27±0.10	0.26±0.10	0.27±0.10	0.29±0.11	0.168
SR	0.01±0.05	0.02±0.05	0.01±0.03	0.01±0.06	0.570
Spherical HOA RMS§¶	0.10±0.05	0.08±0.05	0.10±0.05	0.10±0.05	<0.001*
$Z_{4}^{0}$ §	−0.09±0.05	−0.08±0.05	−0.09±0.05	−0.10±0.05	0.001*
$Z_{6}^{0}$	0.00±0.02	0.00±0.02	0.00±0.02	0.00±0.02	0.528
Comatic HOA RMS	0.15±0.09	0.14±0.08	0.15±0.08	0.16±0.10	0.277
$Z_{3}^{-1}$	−0.05±0.14	−0.04±0.13	−0.06±0.13	−0.06±0.15	0.339
$Z_{3}^{1}$	0.02±0.08	0.02±0.08	0.03±0.08	0.02±0.09	0.741
$Z_{5}^{-1}$	−0.01±0.03	0.00±0.03	−0.01±0.03	−0.01±0.03	0.049*
$Z_{5}^{1}$	0.00±0.01	0.00±0.02	0.00±0.02	0.00±0.01	0.601
Trefoil HOA RMS	0.14±0.08	0.14±0.08	0.14±0.07	0.15±0.08	0.215
$Z_{3}^{-3}$	0.05±0.11	0.04±0.11	0.05±0.11	0.06±0.12	0.545
$Z_{3}^{3}$	0.03±0.08	0.04±0.09	0.03±0.08	0.02±0.09	0.097
$Z_{5}^{-3}$	−0.01±0.03	−0.01±0.04	−0.01±0.03	0.00±0.03	0.022*
$Z_{5}^{3}$	0.00±0.02	0.00±0.02	0.01±0.02	0.01±0.03	0.708

The t-tests with Bonferroni Correction was used for the comparison between subgroups.

The bold values were those P values <0.05.

*P<0.05 was considered statistically significant.

†The analysis of variance test was used for the comparison of spherical equivalent, axial length and HOA among three age groups.

‡P<0.0167 for comparison between the age (13–15 years) group and age (16–18 years) group.

§P<0.0167 for comparison between the age ≤12 years group and the age (16–18 years) group.

¶P<0.0167 for comparison between the age ≤12 years group and the age (13–15 years) group.

HOA, higher-order aberration; RMS, root mean square; SR, strehl ratio.

Of the entire study group, 209 (45.6%) were boys, and 249 (54.4%) were girls. The mean AL (27.14±0.98 mm vs 26.50±0.93 mm), ocular total HOA RMS (0.26±0.13 µm vs 0.25±0.09 µm) and corneal total HOA RMS (0.29±0.11 µm vs 0.26±0.09 µm) for boys were larger than those for girls (p<0.001, p=0.008, and p=0.014, respectively). The ocular and corneal primary spherical aberrations ( $Z_{4}^{0}$ ) for boys were larger than those for girls. The other tested parameters did not show any statistically significant differences between the genders.

The numbers of children from the ≤12, 13–15 and 16–18 years age groups were 146, 209 and 103, respectively. ALs and IPDs were significantly longer in the older age group, while the SEs of the older group worsened (table 1). Corneal spherical HOA RMS and ocular trefoil HOA RMS increased in the older group, while changes in other HOA parameters and SR were not significant. Same trends were found in the RMS of individual Zernike terms (online supplemental table 1). When comparing the individual Zernike coefficients, ocular $Z_{4}^{0}$ were found to have increased from 0.00±0.08 µm to 0.02±0.08 µm (p=0.011), while corneal $Z_{4}^{0}$ decreased from −0.08±0.05 µm to −0.10±0.05 µm (p=0.001). Other individual Zernike coefficients did not share this trend when comparing ocular and corneal groups. When taking these two factors together, we found that boys had a significantly higher level of ocular $Z_{4}^{0}$ than girls at ≤12 years (p=0.009). No significant difference was found in the other HOAs or in other age groups.

### Changes in demographics and HOAs in highly myopic subjects after one year

Whether changes in spherical aberration in the 1-year follow-up presented in the same way as it did between large age gaps was then calculated. Table 3 summarises the demographics and ocular HOA components at each visit (note that only 99 data points available for all visits (baseline, first year) are presented) in high-myopia patients. The SE decreased by −0.76±0.46 D (p<0.001), with astigmatism decreasing by −0.31±0.34 D (p<0.001), while the IPD increased by 0.66±1.39 mm (p<0.001) and the AL increased by 0.23±0.14 mm (p<0.001) after 1 year. The RMS of individual ocular and corneal HOA were analysed in online supplemental table 2.

**Table 3:** Demographics and HOAs of the pooled population at different visits and comparison with an early study

Parameters	Baseline (n=99)	First year (n=99)	Hiraoka’s study (n=71)	P value*
Age, years	12.54±2.53	13.54±2.53	9.20±1.60	<0.001
SE, D	−7.34±1.71	−8.10±1.75	−2.73±0.74	<0.001
Astigmatism, D	−1.26±0.96	−1.57±1.00		
AL, mm	26.46±0.90	26.69±0.89	24.58±0.73	<0.001
Ocular, μm				
Total HOA RMS	0.25±0.10	0.25±0.10	0.35±0.13	<0.001
SR	0.03±0.09	0.02±0.04		
Spherical HOA RMS	0.06±0.05	0.06±0.04		
$Z_{4}^{0}$	0.01±0.08	0.01±0.07	0.07±0.12	<0.001
$Z_{6}^{0}$	−0.01±0.01†	−0.01±0.01†		
Comatic HOA RMS	0.18±0.10	0.17±0.10		
$Z_{3}^{-1}$	0.11±0.14†	0.12±0.13†	0.11±0.19	1.000
$Z_{3}^{1}$	−0.08±0.07†	0.04±0.07†	0.01±0.11	<0.001
$Z_{5}^{-1}$	0.02±0.02	0.01±0.03		
$Z_{5}^{1}$	0.01±0.01†	0.00±0.01†		
Trefoil HOA RMS	0.12±0.07	0.12±0.07		
$Z_{3}^{-3}$	−0.03±0.10†	−0.02±0.10†	−0.05±0.12	0.236
$Z_{3}^{3}$	−0.02±0.09	−0.03±0.09	0.00±0.12	0.213
$Z_{5}^{-3}$	0.01±0.02†	0.00±0.01†		
$Z_{5}^{3}$	0.00±0.01	0.00±0.01		
Corneal, μm				
Total HOA RMS	0.29±0.11†	0.26±0.11†	0.40±0.11	<0.001
SR	0.01±0.03†	0.02±0.03†		
Spherical HOA RMS	0.09±0.04†	0.10±0.05†		
$Z_{4}^{0}$	−0.09±0.04†	−0.10±0.05†	0.22±0.08	<0.001
$Z_{6}^{0}$	0.00±0.02	0.01±0.02		
Comatic HOA RMS	0.15±0.09†	0.13±0.08†		
$Z_{3}^{-1}$	−0.03±0.13†	−0.07±0.12†	−0.01±0.17	0.383
$Z_{3}^{1}$	0.08±0.07†	−0.01±0.06†	−0.15±0.10	<0.001
$Z_{5}^{-1}$	0.00±0.03	−0.01±0.02		
$Z_{5}^{1}$	0.00±0.01†	0.01±0.01†		
Trefoil HOA RMS	0.17±0.08†	0.14±0.09†		
$Z_{3}^{-3}$	0.11±0.11†	0.03±0.12†	−0.04±0.08	<0.001
$Z_{3}^{3}$	0.05±0.08†	0.03±0.10†	−0.02±0.08	<0.001
$Z_{5}^{-3}$	−0.03±0.03†	0.00±0.03†		
$Z_{5}^{3}$	0.00±0.03	0.01±0.03		

*The t-tests with Bonferroni Correction was used for the comparison between our study and Hiraoka’s study.

†P<0.05 was considered statistically significant for the paired t-test used for the comparison between different visits.

AL, axial length; HOA, higher-order aberration; RMS, root mean square; SE, spherical equivalent; SR, strehl ratio.

**Figure 1:**
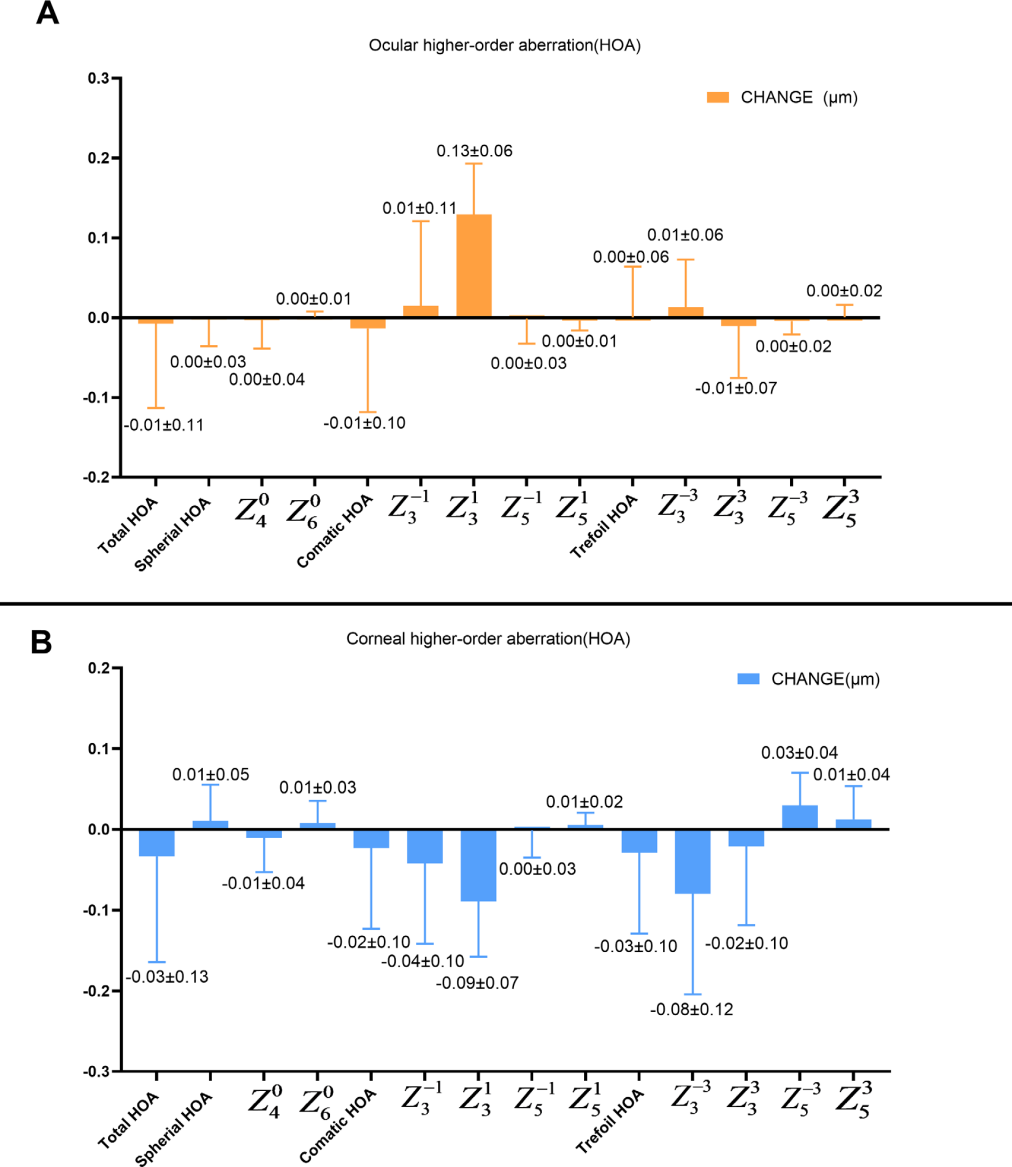
One-year changes in different HOA components. (A) Ocular HOA components. (B) Corneal HOA components.The orange symbols represent ocluar data, and the blue symbols represent corneal data.

The RMS values of corneal total, comatic and trefoil HOAs decreased by −0.03±0.13 µm, −0.02±0.10 µm, and −0.03±0.10 µm, respectively (all p<0.05), while spherical HOAs increased by 0.01±0.05 µm (p=0.025). Ocular HOA RMS and SR was stable with no further significant changes observed at the 1-year visit. When compared with an early study of moderately myopic individuals,^13^ most of the parameters were significantly different, except for ocular and corneal primary vertical comatic aberrations ( $Z_{3}^{-1}$ ). Figure 1 shows the preliminary analysis of ocular and corneal HOA changes after 1 year. Spherical and horizontal comatic aberrations exhibited significantly different changes after 1 year and were significantly lower than the baseline data from moderate myopia presented in an early study (all p<0.05).^13^ Marked changes in ocular primary horizontal comatic aberrations ( $Z_{3}^{1}$ ) (from −0.08±0.07 µm to 0.04±0.07 µm) and corneal $Z_{3}^{1}$ (from 0.08±0.07 µm to −0.01±0.06 µm) were observed in our high-myopia cohort. Same trends were found in the RMS of individual Zernike terms (online supplemental table 2).

### Association between 1-year AL and its elongation with HOA changes in highly myopic subjects

From the linear mixed model analyses (model 1, table 4), as expected, AL was shorter in girls (0.343 mm shorter, p=0.002), increased with IPD (p<0.001), and less so in highly myopic subjects with lower SE (ie, 0.289 mm shorter per 1 D of SER, p<0.001). After adjusting for RMS and SR values, lower RMS values for ocular spherical HOA were associated with longer AL (spherical HOA RMS: β=−3.603 mm/µm, p=0.026, model 1). When investigating the influence of individual Zernike coefficients, a longer AL was associated with a lower level of positive spherical aberration ( $Z_{4}^{0}$ ) (β=−2.777 mm/µm, p<0.001, model 2) and positive comatic aberration ( $Z_{5}^{1}$ ) (β =−11.846 mm/µm, p=0.019, model 3). No associations between the RMS of trefoil aberrations or individual trefoil Zernike terms and AL were observed (all p>0.05). Moreover, no association between corneal HOA and AL was observed (all p>0.05). These findings were not found in the previous study of moderate myopia.^13^

**Table 4:** Statistically significant fixed effects from linear mixed models of the influences of HOA component changes on axial length*

Model	Parameters	β	P value
Model 1 Ocular HOA RMS (total, spherical, comatic and trefoil) and SR	Intercept	19.772	<0.001
	Gender†	−0.343	0.002
	IPD	0.066	<0.001
	SE	−0.289	<0.001
	Spherical HOA	−3.603	0.026
Model 2 Ocular HOA Spherical Zernike components	Intercept	19.408	<0.001
	ln (Age)	0.646	0.036
	Gender†	−0.415	<0.001
	IPD	0.063	<0.001
	SE	−0.269	<0.001
	$Z_{4}^{0}$	−2.777	<0.001
Model 3 Ocular HOA Comatic Zernike components	Intercept	20.281	<0.001
	Gender†	−0.363	0.001
	IPD	0.061	<0.001
	SE	−0.295	<0.001
	$Z_{5}^{1}$	−11.846	0.019

*Parameters and interactions showing statistical insignificance are not shown, for example, ocular Zernike terms and all corneal HOA terms. A p<0.05 was considered statistically significant.

†Parameter estimated for girls.

HOA, higher-order aberration; IPD, inter pupillary distance; RMS, root mean square; SE, spherical equivalent; SR, strehl ratio; β, parameter estimates.

## Discussion

Our study investigated the baseline characteristics of ocular and corneal HOAs as well as their longitudinal changes in 1 year in highly myopic Chinese children and adolescents. The results of this study showed that almost all ocular HOAs and few corneal HOAs were significantly different among different age groups in highly myopic patients. In addition, although primary horizontal comatic aberration changed most, 1-year AL growth was positively associated with a higher level of ocular secondary horizontal coma after adjusting for known confounding variables (age, gender, SE, astigmatism and IPD).

In our study, the absolute values of ocular and corneal primary spherical aberrations displayed an increasing trend in line with the growth of age groups as well as changes after 1 year. Lens power plays a key role in the spherical aberration changes that lead to the stabilisation of corneal power.^34^ A positive change in the primary ocular spherical aberration ( $Z_{4}^{0}$ ) from infancy (negative) through early childhood (positive) has been reported in various studies.^15 35^ In a previous study, differences in lens power showed a decreasing tendency with increasing age and were greater in participants younger than 9 years old.^36^ Therefore, similar trends in primary spherical aberration changes in high myopia might indicate main changes from the alignment of ocular components (eg, lens), as the corneal spherical aberration (representing the power of cornea) presented a decreasing trend. Boys had a larger primary spherical aberration value than girls at <12 years of age. In addition, boys had a longer AL and less lens power than girls.^37^ The difference in lens power between boys and girls was not related to lens thickness.^38^ The same phenomenon had been found in comparison with moderate myopia, which had more positive lens power, thus primary ocular spherical aberration were higher in moderate myopia. Taken together, we hypothesised that primary ocular spherical aberration mainly represents lens power.

The ocular and corneal primary horizontal comatic aberrations exhibited the highest change after 1 year. In our study groups, all astigmatic participants had with-the-rule astigmatism with negative cylinders of 180°±20°. This could explain why comatic changes were mainly in the horizontal direction and the difference in comatic aberrations between our study and the early moderate myopia study. This finding is consistent with that of Philip, who reported that comatic aberration was the only HOA component that increased significantly from baseline in the myopic change group.^27^

Axial elongation in 1 year was followed by a decrease in ocular comatic HOA RMS, a finding that differed from other studies in which AL changes were only significantly related to spherical aberrations.^14 24 39^ Further analysis of both ocular and corneal comatic Zernike components showed that ocular secondary horizontal coma ( $Z_{5}^{1}$ ) was the only individual comatic aberration that was positively correlated with longer AL at the 1-year follow-up. This 1-year shift in comatic aberrations might be caused by intrinsic factors such as the shape of the corneal and crystalline lens surfaces or the tilt and decentration of the optical components.^27 40^ However, subjects in our cohorts had longer (highly myopic) eyes; thus, the compensatory ability of the lens power might have been limited.^36^ Above all, ocular secondary horizontal coma was not affected by lens power but by decentration of the optical components.

No significant association was observed between Strehl Ratio changes after 1 year and development and progression of myopia among highly myopic eyes, which was in line with an early study in emmetropic eyes.^27^ This suggests that retinal image quality change caused by HOA does not trigger ocular growth.

Our study had some limitations. First, the relatively small sample size were not large enough to investigate the effect of HOAs on axial eye growth in different refractive error and age groups (including adults).^41 42^ Previous study also found 9 years old was a turning point in lens power.^36^ We could not figure out if things will be the same in HOA as only 18 children were under 9 years old in this study. Larger population studies are required to prove the influence of HOA on the development of myopia. Moreover, accommodative response or habitual correction were not evaluated in our study, which might cause changes in HOAs.^43^ The fixed 5 mm pupil chosen in our study is consistent with pupil diameters under low luminance during the examination.^44^ Additionally, measures of HOA components under habitual conditions without cycloplegia as well as other confounding factors, such as parental myopia, outdoor activities and living conditions, may provide further insights into the relationships between eye optics and axial growth.^26 28 29^ HOA components were measured via Shack Hartmann aberrometer, thus our results could not be directly applied to instruments using other types of aberrometer, that is, ray-tracing.^45^ Furthermore, while a significant association was observed between various HOA components and axial elongation after controlling for confounding variables, this relationship does not necessarily infer a causal relationship between the HOA component and axial eye growth. Besides, there was a relationship between relative peripheral refractive error and HOA in axial elongation.^46^ Comatic aberration might be a bridge connected these two mechanisms.^47^ Further research should be done to prove this conjecture.

In conclusion, ocular comatic HOAs were negatively associated with axial elongation in myopic schoolchildren and adolescents. Higher levels of ocular secondary horizontal comatic aberrations were the most relevant factor correlated with faster axial elongation in high myopia after adjusting for confounders, such as age, refraction and IPD. Changes in ocular HOAs, notably horizontal comatic aberrations, besides spherical aberrations may take part in refractive development of high myopia.

## Data Availability

Data are available on reasonable request.
