# Supplementary material for: Higher-order aberrations and their association with axial elongation in highly myopic children and adolescents
Source: Br J Ophthalmol. 2022 Jan 13;107(6):862–8. doi: 10.1136/bjophthalmol-2021-319769 (PMC10314045; doi:10.1136/bjophthalmol-2021-319769)
Supplement: Supplementary data [file bjophthalmol-2021-319769supp001.pdf]

Supplemental Material

The Root Mean Square of individual Ocular and Corneal Higher-order Aberrations of Pooled Population and Difference Among Different Age Groups at baseline was listed in Supplementary Table 1. When comparing the individual Zernike coefficients, RMS of Ocular and corneal spherical HOA increased in the older group. RMS of ocular primary vertical comatic aberrations (Z_3^{-1}) were found to have increased from 0.09μm to 0.21μm, while corneal primary vertical comatic aberrations (Z_3^{-1}) increased from 0.14μm to 0.17μm with age. RMS of ocular primary vertical trefoil aberrations (Z_3^{-3}) were found to have increased from 0.10μm to 0.12μm, while corneal primary vertical trefoil aberrations (Z_3^{-3}) increased from 0.12μm to 0.13μm with age. These findings were in line with the trends in individual Zernike terms evaluated in the forms of mean values.

SUPPLEMENTARY TABLE 1 RMS of individual Ocular and Corneal Higher-order Aberrations of Pooled Population and Difference Among Different Age Groups at baseline

Parameters	Total (n=458)	Age, years		
		≤12 (n=146)	13-15 (n=209)	16-18 (n=103)
Ocular, μm				
Z_4^0	0.08	0.08	0.08	0.08
Z_6^0	0.01	0.01	0.01	0.01
Z_3^{-1}	0.19	0.18	0.18	0.21
Z_3^1	0.09	0.09	0.09	0.09
Z_5^{-1}	0.03	0.02	0.03	0.03
Z_5^1	0.01	0.01	0.01	0.01

Z_3^{-3}	0.10	0.10	0.09	0.12
Z_3^{3}	0.08	0.09	0.08	0.08
Z_5^{-3}	0.02	0.02	0.02	0.02
Z_5^{3}	0.01	0.01	0.01	0.01
Corneal, μm				
Z_4^{0}	0.11	0.09	0.11	0.11
Z_6^{0}	0.02	0.02	0.02	0.02
Z_3^{-1}	0.15	0.14	0.14	0.17
Z_3^{1}	0.08	0.08	0.08	0.09
Z_5^{-1}	0.03	0.03	0.03	0.03
Z_5^{1}	0.02	0.02	0.02	0.01
Z_3^{-3}	0.12	0.12	0.12	0.13
Z_3^{3}	0.09	0.10	0.09	0.09
Z_5^{-3}	0.04	0.04	0.03	0.03
Z_5^{3}	0.03	0.02	0.02	0.03

Abbreviations: RMS: Root Mean Square;

Marked changes in ocular primary horizontal comatic aberrations (Z_3^1) (from 0.11 μm to 0.08 μm) and corneal primary horizontal comatic aberrations (Z_3^1) (from 0.11 μm to 0.06 μm) were observed in our high-myopia cohort, which was also in line with the mean value changes of individual Zernike terms (Supplementary Table 2) .

SUPPLEMENTARY TABLE 2 RMS of Higher-order Aberrations of the Pooled Population at Different Visits

**Parameters**	**Baseline (n=99)**	**First year (n=99)**	**Baseline (n=99)**	**First year (n=99)**
	Ocular, μm	Ocular, μm	Corneal, μm	Corneal, μm
Z_4^0	0.08	0.07	0.10	0.11
Z_6^0	0.01	0.01	0.02	0.02
Z_3^{-1}	0.17	0.18	0.13	0.14
Z_3^1	0.11	0.08	0.11	0.06
Z_5^{-1}	0.03	0.03	0.03	0.02
Z_5^1	0.01	0.01	0.01	0.02
Z_3^{-3}	0.11	0.10	0.15	0.12
Z_3^3	0.09	0.10	0.10	0.10
Z_5^{-3}	0.02	0.01	0.04	0.03
Z_5^3	0.01	0.01	0.03	0.03

Abbreviations: RMS: Root Mean Square
